# Using local ecological knowledge to monitor threatened Mekong megafauna in Lao PDR

**DOI:** 10.1371/journal.pone.0183247

**Published:** 2017-08-18

**Authors:** Thomas N. E. Gray, Amphone Phommachak, Kongseng Vannachomchan, Francois Guegan

**Affiliations:** 1 WWF Greater Mekong, 21, Boeun Keng Kang 1, Phnom Penh, Cambodia; 2 WWF-Laos, Unit 05, Saylom Village, Vientiane, Lao Democratic People’s Republic; Pacific Northwest National Laboratory, UNITED STATES

## Abstract

Pressures on freshwater biodiversity in Southeast Asia are accelerating yet the status and conservation needs of many of the region’s threatened fish species are unclear. This impacts the ability to implement conservation activities and to understand the effects of infrastructure developments and other hydrological changes. We used Local Ecological Knowledge from fishing communities on the Mekong River in the Siphandone waterscape, Lao PDR to estimate mean and mode last capture dates of eight rare or culturally significant fish species in order to provide conservation monitoring baselines. One hundred and twenty fishermen, from six villages, were interviewed. All eight species had been captured, by at least one of the interviewees, within the waterscape within the past year. However the mean and mode last capture dates varied between the species. Larger species, and those with higher Red List threat status, were caught less recently than smaller species of less conservation concern. The status of the Critically Endangered *Pangasius sanitwongsei* (mean last capture date 116.4 months) is particularly worrying suggesting severe population decline although cultural issues may have caused this species to have been under-reported. This highlights that studies making use of Local Ecological Knowledge need to understand the cultural background and context from which data is collected. Nevertheless we recommend our approach, of stratified random interviews to establish mean last capture dates, may be an effective methodology for monitoring freshwater fish species of conservation concern within artisanal fisheries. If fishing effort remains relatively constant, or if changes in fishing effort are accounted for, differences over time in mean last capture dates are likely to represent changes in the status of species. We plan to repeat our interview surveys within the waterscape as part of a long-term fish-monitoring program.

## Introduction

Freshwater habitats are amongst the most endangered ecosystems globally and are experiencing biodiversity loss at greater rates that terrestrial equivalents [1–5]. The pressures on freshwater ecosystems worldwide are encapsulated by the Mekong: the largest river in Asia and ranked amongst the top three rivers globally in terms of fish diversity [5,6]. The Mekong also supports a unique assemblage of freshwater megafauna (species >100-kg and >180-cm long) including four of the six largest freshwater species globally. Three of these species, Mekong giant catfish *Pangasianodon gigas*, giant carp *Catlocarpio siamensis*, and dog-eating catfish *Pangasius sanitwongsei*, are IUCN listed as Critically Endangered largely as a result of declines driven by unsustainable levels of exploitation [7,8]. Additional threats to Mekong biodiversity include extensive planned infrastructure developments that are likely to impact habitat quality and cause fragmentation particularly for migratory species such as *Pangasianodon gigas* and *Pangasius sanitwongsei* (henceforth mega-catfish) [4,9]. Twelve large hydropower dams are proposed on the un-dammed lower and middle mainstream Mekong River in the Lao PDR, Thailand, and Cambodia and these would have implications for both human livelihoods and biodiversity [10–13]. Basin-wide hydrological changes caused by increases in levels of annual glacier melt [14,15] and the impacts of pollution from industrial agriculture (e.g. Economic Land Concession in Cambodia; [16,17]) are likely to create additional stresses to freshwater biodiversity in the Mekong. However a lack of knowledge of the status, distribution, and trends of Mekong megafauna is impacting the ability to implement, and monitor success of, conservation activities for these species and understand the impacts of hydropower development and other environmental changes.

Local Ecological Knowledge (LEK), the knowledge of local communities obtained from interactions with their environment, is increasingly used by conservationists to provide information on distribution [18], trends [19], and abundance [20] of threatened species. For species that are large and distinctive, or have high socio-economic or cultural importance, local communities can often provide information on the status or last occurrence of threatened species. In such circumstances targeted community interviews may represent a robust and cost-effective method for collecting data [21]. For example by recording the last known sighting date of various terrestrial ungulates in Vietnam and the Lao PDR Turvey *et al*., [22] were able to generate information on the current status of the mammal community including saola *Pseudoryx nghetinhensis*, one of the world's rarest mammals. Given their intense interactions with the natural environment, and the high economic and cultural value of many of the species they catch, LEK from fishing communities is widely used in fisheries management and has been shown to provide largely robust data [23,24].

To address the urgent need to acquire baseline knowledge on the status of threatened Mekong freshwater fish species we conducted targeted interviews with local fishermen to determine last catch dates for Mekong megafauna, and a number of other threatened species, from the Siphandone waterscape in southern Lao PDR. Recent sampling using environmental DNA (eDNA) has demonstrated this area supports amongst the highest diversity of fish species from the Mekong basin [7] however the waterscape is threatened due to infrastructure development [25]. The aims of the study were to provide a baseline of mean and mode last capture dates for a number of culturally and ecologically significant species in order to provide a baseline against which either positive (e.g. through the implementation of conservation activities) or negative (e.g. through the impact of infrastructure development) change can be measured.

## Materials and methods

### Study area

The Siphandone waterscape (Approx. 13^o^58’N 105^o^54’E) is a mosaic of islands and channels in the mainstream Mekong around the Khone falls on the border of the Lao PDR and Cambodia. The Khone falls represent a biogeographic boundary between the Lower and Middle Mekong and many of the waterfalls are impassable barriers to fish whilst other channels and rapids are passages for strongly migratory species. The waterscape forms one of the most important wild-capture riverine fisheries in tropical Asia and >90% of families participate in artisanal fisheries [25–27]. In this paper the Siphandone waterscape refers to areas on, or alongside. the Mekong in Khong and Mounlapamok districts, Champasak province, Lao PDR.

### Local ecological knowledge surveys

We conducted targeted random interviews in fishing communities in six villages within the Siphandone waterscape in September 2014. The six survey villages (Table 1) were randomly selected from a geographically stratified list of all villages (n = 80) within the waterscape. The selection of villages was stratified geographically to ensure representative sampling of island and mainland villages and a consistent north-to-south spread of communities surveyed.

**Table 1:** Population, and number of interviewees within target survey villages in Siphandone, Lao PDR. Villages located in Khong district on islands within the Mekong.

Survey Village	District	Population	Number of interviewees
Hangsadam	Khong	529	20
Lopadikhonnoi	Khong	1,458	20
Donphapeng	Khong	174	20
Nadi	Mounlapamok	1,496	20
Veunkhaen	Mounlapamok	3,138	20
Veun	Mounlapamok	450	20

Within each village a list of all families involved in fishing was obtained from district authorities and twenty families selected using random number generator in Microsoft Excel. The principal fisher from each selected family (who was always male) was interviewed on a one-to-one basis by a native Lao speaker (KV) following a standard questionnaire containing descriptive, structured, and contrast questions that took 45–70 minutes to complete.

As part of a wider series of questions, interviewees were asked whether they knew of, and had personally caught, each of eight species of Mekong fish (henceforth focal species): three species of globally threatened Mekong megafauna (i.e. which reach maximum size of >100-kg), three additional globally threatened and culturally relevant species, and two species selected to represent common and widely known fish as ‘control’ species (Table 2). All of these species are important for both commercial and subsistence fisheries within the Mekong with the *Probarbus* fisheries of Siphandone particularly significant [28]. During project design *Probarbus jullieni* was identified as one of the focal species however data collection did not always differentiate between *P*. *jullieni* and *P*. *labeamajor* and thus, conservatively, we interpret our results as referring to *Probarbus* ssp. It is also worth noting that *P*. *labeamajor* may be more of a habitat specialist and thus may not occur in all locations in which *P*. *jullieni* is found.

**Table 2:** Focal species of Mekong fish for which last capture dates established from interviews with fishermen in Siphandone, Lao PDR.

Species and Family	English name	Lao name	~ Max weight (kg)	IUCN Status
Pangasianodon gigas*, Chevey 1931; Pangasiidae	Mekong giant catfish	Pa beuk	350	CR
Pangasius sanitwongsei*, Smith 1931; Pangasiidae	Dog-eating catfish	Pa leum	300	CR
Himantura polylepis*, Bleeker 1852; Dasyatidae	Giant freshwater whipray	Pa fa lay yai	400	EN
Dasyatis laosensis*, Roberts & Karnasuta 1987; Dasyatidae	Mekong freshwater stingray	Pa fa lay noy	30	EN
*Probarbus jullieni*, Sauvage 1880 & *P*. labeamajor* Roberts 1992; Cyprinidae	Jullien’s golden carp / thick-lipped barb	Pa eun ta deang / Pa eun ta leung	70	EN
Bangana behri*, Fowler 1937; Cyprinidae	n/a	Pa wa houa nano	10	VU
Hemibagrus spilopterus*, Ng & Rainboth 1999; Bagridae	Mekong red-tail catfish	Pa kot	5	LC
*Barbonymus gonionotus*, Bleeker 1849; Cyprinidae	Silver barb	Pa pak	5	LC

IUCN status—CR–Critically Endangered, EN–Endangered, VU–Vulnerable, LC–Least Concern.

Mekong megafauna highlighted in bold; endemic species to Mekong basin indicated by asterix (*), control species underlined.

Fish species were identified using a set of colour photographs and Lao language names for each species. Interviewees were also questioned about the behavior and habitat preferences of each species (e.g. months they breed, migratory status, preference for deep pools vs rapids) to further reduce the possibility of erroneous identification. If identification was uncertain, inconsistent data was provided, or respondents did not appear to know the species, data was not used. This resulted in discarded data from between one (*Hemibagrus spilopterus*) and 79 (*Pangasianodon gigas*) interviewees (S2 Table). Nevertheless the principal interviewer (KV) had limited knowledge of the natural history of Siphandone fish species prior to commencing this study and is a native of Bokeo province, in northern Lao PDR. As such we cannot guarantee that a number of misidentifications have not entered the dataset.

If respondents had personally caught the species they were asked to give the most recent date (month and year) in which they caught it. Respondents were also asked to give a numeric ranking to their perception of the status of each species 20 years ago, 15 years ago, 5 years ago, 2 years ago and at the current time. Respondents were asked to score each species for each period as 1: common 2: uncommon 3: rare or 4: extinct.

The majority of interviewees (79% of responses) gave a specific month and year for the most recent time they had caught each species; these were converted into months prior to the interview i.e. a last capture in September 2013 equated to 12 months. If only a year was given (12% of responses) the last capture date was calculated assuming the capture at the mid-point of the year i.e. a last capture year of 2010 was converted to June 2010 and thus equated to 51 months. When interviewees had previously caught the species but could not give a year of last capture (9% of responses) data was not used for analysis. The mean last capture month, and corresponding 95% confidence intervals, were calculated for each species using Package ‘lsr’ which calculates confidence intervals for the mean of a normally-distributed variable [29], in R software [30]. The mode and minimum last capture month were also calculated for each species.

#### Ethics statement

The study design and interview questions used were reviewed by both the conservation and ethics committee of WWF Greater Mekong and the Ministry of Agriculture and Forestry of the Lao PDR government. As the fieldwork took place on government land no specific permission was required for fieldwork beyond that covered under the Memorandum of Understanding between WWF Greater Mekong and the government of Lao PDR. All participation in interviews was voluntary and data held anonymously. Despite being based on fish-catch data from artisanal fishermen no animals were caught or harmed in any way as a direct result of this study. As such animal research ethics committee approval was not deemed necessary.

## Results

A total of 120 fishermen (all male) were interviewed from the six fishing communities (Table 1). Interviewees were aged between 22 and 73 years (mean 44 ± SD 11) and had been fishing for a mean of 27 ± SD 12 (range 4–58) years. The age structure of fishermen and years fishing were similar across the six communities (S1 Table). All interviewees were currently active fishermen and estimated they fished for between 4 and 70 (mean 23 ± SD 13) hours per week. However weekly hours spent fishing differed substantially between the villages being highest in Donphapheng (mean 39 ± SD 13.2 hours per week) and lowest in Veunkhaen and Veun (mean 14 ± SD 9 hours per week). Fishermen reported using at least 13 types of fishing gear and fishing traps of which gill-nets were the most widely used (98% of fishermen); ‘li’ traps, a specific fishing technique used in the Siphandone waterscape [28] were used by approximately 30% of interviewed fishermen.

Six of the target fish species—*Himantura Polylepsis*, *Dasyatis laosensis*, *Probarbus* spp., *Bangana behri*, *Hemibargus spilopterus*, and *Barbonymus gonionotus*—had been caught by >83% of the interviewees with a last catch-date (either month or year) provided by between 85 (*Dasyatis laosensis*) and 115 (*Hemibargus spilopterus*) fishermen (S2 Table). In contrast the two mega-catfish were unambiguously known by less than half of the interviewed fishermen and had been caught by 24 (20%; *Pangasianodon gigas)* and 43 (36%; *Pangasius sanitwongsei)* interviewees. Last catch-dates (month or year) for these two species were obtained from 19 (*Pangasianodon gigas*) and 34 (*Pangasius sanitwongsei*) fishermen (S2 Table). Unlike the more commonly caught species there was heterogeneity in capture rates of the two mega-catfish between villages (S3 Table); for example *Pangasianodon gigas* had been caught by 65% of interviewees in Hangsadam compared to less than 20% in the other five villages (S3 Table).

**Fig 1:**
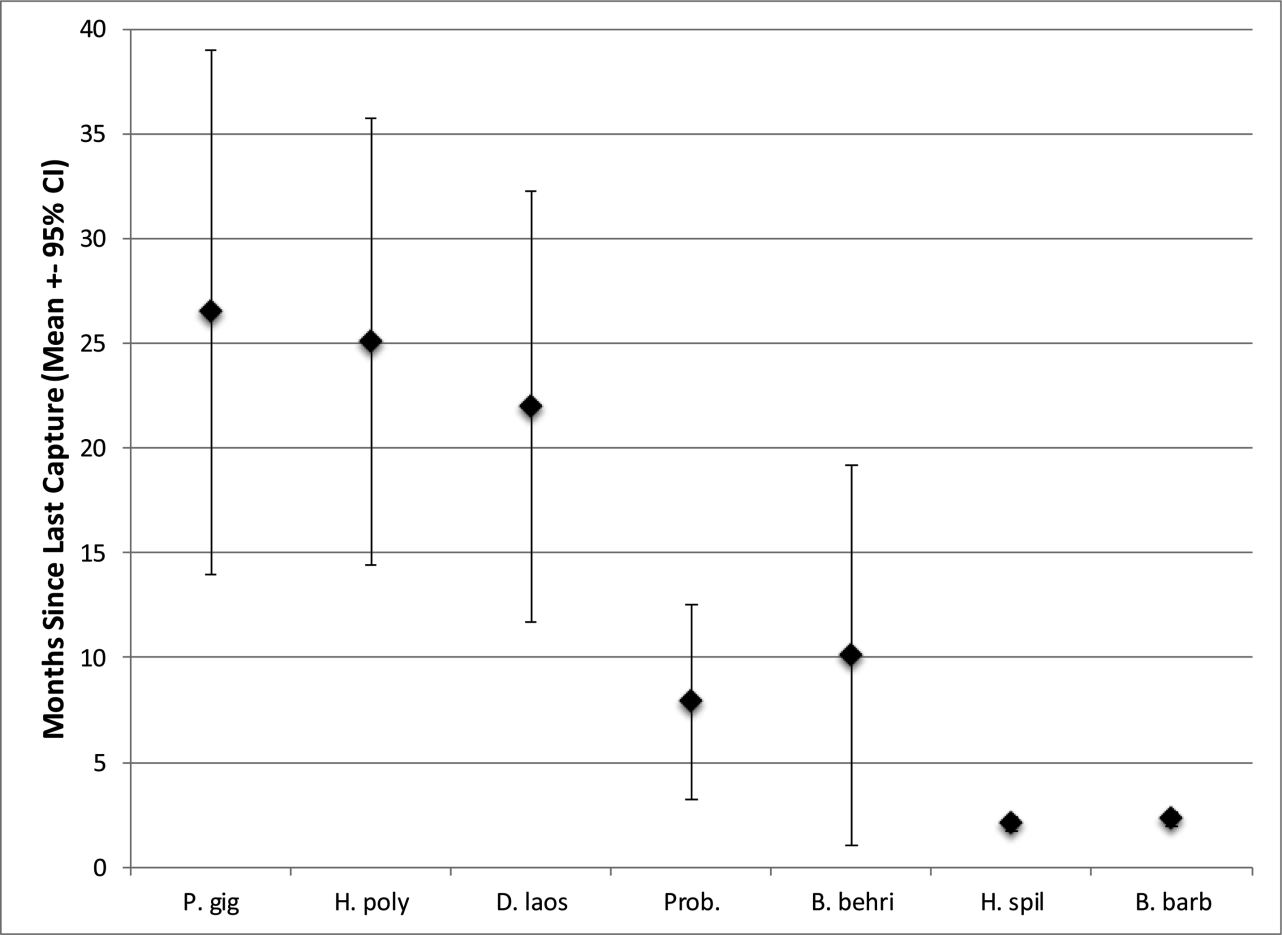
Mean (± 95% confidence interval) last capture date (in months) of focal species from 120 interviewed fishermen in Siphandone, Lao PDR. Species abbreviations: P. gig *Pangasianodon gigas;* H. poly *Himantura polylepis;* D. laos *Dasyatis laosensis;* Prob. *Probarbus spp;* B. behri *Bangana behri;* H. spil *Hemibagrus spilopterus;* B. barb *Barbonymus gonionotus* Note *Pangasius sanitwongsei* not included in the figure due to the species’ large last capture date.

Despite the substantial differences in mean last catch date between the eight species (Table 3; Fig 1) all had been caught by at least one interviewee in 2014 (Table 3) indicating all remain extant within the Siphandone waterscape. However based on mean last capture dates the eight focal species clustered into four distinct groups a) *Pangasius sanitwongsei* with a mean last capture date of just under 10 years ago; b) *Pangasianodon gigas*, *Himantura polylepsis*, and *Dasyatis laosensis* with mean last capture dates of between 22 (1.8 years) and 26.5 (2.2 years) months. However the mode last capture date was considerably higher for *Pangasianodon gigas* (13 months) than the two freshwater rays (1 and 3 months respectively); c) *Probarbus* spp. and *Bangana behri* with mean last capture dates of 8 and 10 months (i.e. within the last year) and mode last capture date of 1 month; d) *Hemibargus spilopterus*, and *Barbonymus gonionotus* with mean last capture dates of 2 months and mode of 1 month.

**Table 3:** Most recent, mode and mean (with 95% confidence interval) last capture dates (in months) of focal species from 120 interviewed fishermen in Siphandone, Lao PDR.

Species	Most recent capture	Mode last capture date	Mean (95% confidence interval) last capture date
*Pangasianodon gigas*	1	13	26.5 (14–39)
*Pangasius sanitwongsei*	1	128	116.4 (63.9–168.8)
*Himantura polylepis*	1	1	25 (14.4–35.8)
*Dasyatis laosensis*	1	3	22 (11.7–32.3)
*Probarbus spp*	1	1	7.9 (3.2–12.5)
*Bangana behri*	1	1	10.1 (1–19.2)
*Hemibagrus spilopterus*	1	1	2.1 (1.1–2.4)
*Barbonymus gonionotus*	1	1	2.3 (1.9–2.6)

**Fig 2:**
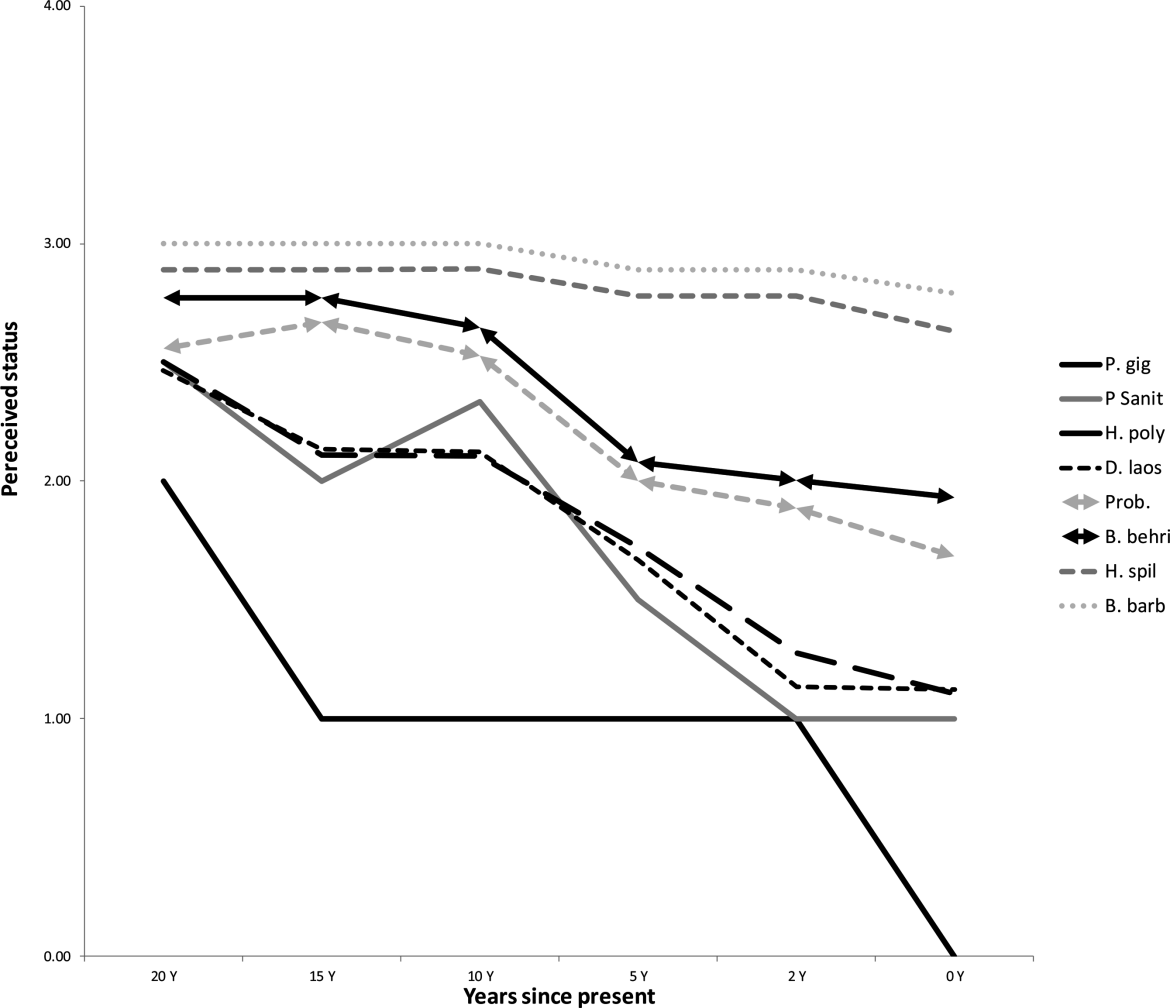
Perceived status of focal species of Mekong freshwater fish from 120 interviewed fishermen in Siphandone, Lao PDR over the past 20 years. 4 common to 0 extinct. Species abbreviations: P. gig *Pangasianodon gigas;* P. sanit *Pangasius sanitwongsei;* H. poly *Himantura polylepis;* D. laos *Dasyatis laosensis;* Prob. *Probarbus spp;* B. behri *Bangana behri;* H. spil *Hemibagrus spilopterus;* B. barb *Barbonymus gonionotus*.

With the exception of *Pangasianodon gigas*, which was described as rare even then, interviewees ascribed all focal species as having similar levels of abundance 20 years ago (Fig 2). Subsequent perceived trends in abundance of the species show similar patterns to mean last catch dates with the exception of an apparent under-estimate of the decline of *Pangasius sanitwongsei*, which was classified as declining at a similar rate to the two freshwater rays, and an over-estimate of the decline of *Pangasianodon gigas* which was rated as ‘extinct’ by 94% interviewees who knew the species. *Probarbus* spp. and *Bangana behri* were both described as having declined, but not at the rate of the mega-catfish and freshwater rays, whilst both *Hemibagrus spilopterus* and *Barbonymus gonionotus* were classified as having similar levels of abundance across the last 20 years (Fig 2).

## Discussion

Tropical freshwater biodiversity is both highly threatened and poorly understood by scientists and conservation policy makers [1,5]. As such Local Ecological Knowledge (LEK) can be a valuable tool for conservationists for example by identifying key sites supporting threatened species [22,31] or estimating species trends and status [20]. We present a novel use of LEK for monitoring the status of threatened and iconic freshwater fish species based on calculating mean last sighting date from stratified random interviews within fishing communities. Last sighting date surveys have previously been used in freshwater Asian environments, including the Yangtze River in China, to calculate mode last capture dates of Cetaceans and other freshwater megafauna and thus document trends and potential extinction dates [19,32]. However few studies have used such data to estimate mean last sighting dates and associated variance. We suggest that this could be a valuable way to incorporate LEK into environmental monitoring. However both government decision-makers and conservation practitioners can be resistant to using findings from LEK to influence policy [33]. This may be particularly problematic in regions, such as Greater Mekong, where government decisions are often non-transparent. As such approaches such as ours, in which LEK can be incorporated into the monitoring programs of international conservation NGOs and government partners, may act as a potential bridge between Western science and LEK [34].

Our data provides monitoring baselines for the status of eight species, six of which are globally threatened, within the Siphandone waterscape, Lao PDR. We believe that the variation between the mean last catch dates within our focal species relates to current abundance levels within the Siphandone waterscape and our results thus indicate the current status of these species. However it is possible that at least some of this variation could be caused by other factors and, as with any biologically sampling process including field-based surveys lead by trained biologists, a number of caveats are attached to both our specific findings and the wider methodology [35].

Errors derived from LEK surveys can arise from inaccurate or untrue responses from interviewees or from the interviewer misunderstanding or misinterpreting the information provided [33,35]. To correctly interpret LEK interview data locally sourced knowledge of the natural history of the target species is important [35]. For example the Lao language name we used for one of the control species *Barbonymus gonionotus* ‘Pa pak’ may be used locally in the Siphandone waterscape to describe multiple species, some of which (e.g. *Hypsibarbus* spp.) may be declining. As *Barbonymus gonionotus* is relatively common and abundant, and was selected in this study as an example control species, this is unlikely to impact our results. However if local names used for one, or more, of our focal species were applied to more than one taxa this would be a more serious issue. This highlights a concern over using LEK for species focussed-conservation efforts: a potential disconnect between locally-derived taxonomy and the western-scientific concepts of species which guide most conservation decision making [36–38].

Cultural and legal issues can also influence responses given by local communities and it is important to understand the cultural context in which interviews take place [33]. For example some communities in the Siphandone waterscape may be reluctant to verbally acknowledge capturing *Pangasius sanitwongsei* because there may be a perception that if the species’ name is spoken they may not be able to catch it again in the future. This could explain why interviewees reported a less significant decline in the perceived abundance of this species, compared with *Pangasianodon gigas*, a finding which contradicted the last-capture date data. Similarly there may have been a reluctance of interviewees to report captures of *Pangasianodon gigas* as this is illegal and community members may be fined for catching or selling the species. Whilst we assured interviewees that all data is anonymous, and would not be passed onto authorities, we cannot guarantee that this did not impact the results.

A number of our focal species are migratory and thus seasonal variation in capture frequency within Siphandone is likely. However given that the variation in last catch date between species was greater than 12-months it is unlikely that the differences we observed are a result of species’ migratory status. This further highlights the need for a strong understanding of the natural history and biology of study species in order to effectively interpret results from surveys using LEK [35]. Target species are also unlikely to be evenly spread across study landscapes and as such stratified sampling of communities, as employed in this study, is recommended.

Variations in inter-species capture probability, for example caused by differences in microhabitat selection and how amendable such microhabitats are to different fishing techniques, could also impact last catch dates between species. Interviewees used 13 different fishing techniques, which are likely to sample the majority of microhabitats within the waterscape and all focal species were culturally or commercially important and thus we assume relatively high capture probabilities. Nevertheless variation in inter-species capture probability could impact the results particularly when comparing between species. We therefore suggest our methodology is most appropriate for temporal monitoring of individual species as part of a long-term monitoring program particularly when surveys return to the same communities. In future it may also be useful to record details of the size of captured fish, and last capture dates of individual fish above threshold sizes, as there are a number of examples globally in which mature large fish are very rare but juveniles are still commonly caught [2]. This modification may make our methodology more sensitive to detecting population declines.

Another possible issue impacting the validity of our, and similar, studies is the accuracy of last capture dates provided by interviewees. Some of the interviewed fisherman gave remarkably precise information–for example a last capture date for *Dasyatis laosensis* from October 1987 from one villager. Exact month of last capture were given for 79% of species captures. Whilst it is impossible to independently validate the accuracy of responses we feel that there is no reason for interviewees to predictably bias their responses (e.g. always give a more recent capture date than what they knew to be the case) and thus we were able to obtain valid information on species status and trends. All interviewees willingly participated in the study and we rarely felt that interviewees were giving deliberately incorrect answers (but see discussion above regarding *Pangasius sanitwongsei*).

Based on the mean and mode last-catch dates, and perceived changes in status over the past 20 years, our focal species clustered into groups that, a-priori, can be defined based on size and IUCN status. Larger species and those with higher IUCN threat level were caught less recently and were perceived to have declined more rapidly than smaller ones and those with lower threat status. These findings generally support the current IUCN listings of these species. The meta-analysis of Olden et al., [39] found body size in marine fish was directly correlated with extinction risk. However in freshwater fish a bimodal pattern existed: both small and large bodied species were likely to be threatened. However freshwater fish species considered important for commercial or subsistence fisheries, as is the case with all our focal species, were much larger than threatened non-commercially fished species [39]. As such the relationship between size, current IUCN global threat status, and status within Siphandone waterscape, as derived from our interviews, is to be expected.

All of the focal species had been caught by at least one interviewee within the past month indicating all remain extant in the Siphandone waterscape. The mode capture date for five of the species, including IUCN threatened *Himantura polylepis*, *Probarbus spp*, and *Bangana behri*, was one month suggesting these species remain regularly caught and that the Siphandone waterscape is significant for their conservation. However the status of *Pangasius sanitwongsei* is of particular concern. Whilst more interviewees had previously caught *Pangasius sanitwongsei* than had caught *Pangasianodon gigas* both the mean and mode last catch date of the former was considerably more recent. This suggests a more extensive decline and emphasizes the perilous conservation status of this species (but see discussion above regarding possible reporting bias due to cultural reasons). *Pangasianodon gigas* was rated as ‘extinct’ by more villagers than all of the other species, including *Pangasius sanitwongsei*, despite the last-capture date being reported as more recent in all villages. *Probarbus spp*, and *Bangana behri* plus the two ‘control’ species, *Hemibagrus spilopterus* and *Barbonymus gonionotus*, were known and caught by >95%, but less than 100%, of interviewed fishermen. It is unclear whether the small number of individuals who had not caught, or did not know of these species, was genuine or a result of unclear species names and or misidentification.

## Conclusions

Monitoring freshwater biodiversity, particularly exploited fish species, is critical for effective conservation management. This is particularly important in rapidly changing river basins such as the Mekong where infrastructure development and climate changes are impacting hydrological processes [5,10,14,15] which in turn are likely to strongly impact globally significant fish populations which are already substantially depleted as a result of historic overexploitation [8]. We suggest our approach, of stratified random interviews to establish mean last capture date, may be an effective and robust methodology for monitoring freshwater fish species of conservation value within artisanal fisheries particularly when interviewers understand the cultural and legal sensitivities around obtaining and analysing Local Ecological Knowledge [35]. If fishing effort remains relatively constant, or if changes in fishing effort / the impact of seasonality are accounted for or modelled as covariates impacting detectability, differences over time in mean last capture dates are likely to represent changes in the status of species. We plan to repeat our interview surveys within the waterscape during 2017 as part of a long-term fish-monitoring program. In addition to providing information on the conservation efficacy of Fish Conservation Zones, and other locally employed management practices, such monitoring can also provide information on the impact of planned hydro-power developments, both within the waterscape and elsewhere on the middle Mekong, on globally threatened fish species.

## Supporting information

S1 Table Mean ± SD and range (in parenthesis) age, years fishing and weekly fishing hours from interviewed fishermen in six survey villages in Siphandone, Lao PDR. (DOC)

S2 Table Knowledge (number of interviewees with % of interviewees in parenthesis) and previous capture experience (number of interviewees with % of interviewees in parenthesis) of focal species of Mekong freshwater fish from 120 interviewed fishermen in Siphandone, Lao PDR. Number of interviewees who provided last catch dates used for analysis indicated including number, in parenthesis, providing a month and year of last capture.
(DOC)

S3 Table Knowledge (number of interviewees with % of interviewees in parenthesis) and previous capture experience (number of interviewees with % of interviewees in parenthesis) of *Pangasianodon gigas* and *Pangasius sanitwongsei* by interviewed fishermen in six survey villages (n = 20 in each village) in Siphandone, Lao PDR. (DOC)

S4 Table Translated interview form used for the study. (DOC)

## References

[1] Dudgeon D, Arthington AH, Gessner MO, Kawabata ZI, Knowler DJ, Lévêque C et al Freshwater biodiversity: importance, threats, status and conservation challenges. Biol. Rev. 2006; 81: 163–182 doi: 10.1017/S1464793105006950 16336747 10.1017/S1464793105006950

[2] Mota M, Sousa R, Araújo J, Braga C, Antunes C. Ecology and conservation of freshwater fish: time to act for a more effective management. Ecol Freshw Fish: 2014;23, 111–113.

[3] Vörösmarty CJ, McIntyre PB, Gessner MO, Dudgeon D, Prusevich A, Green P et al Global threats to human water security and river biodiversity. Nature: 2010; 467, 555–561. doi: 10.1038/nature09440 20882010 10.1038/nature09440

[4] He F, Zarfl C, Bremerich V, Henshaw A, Darwall W, Tockner K et al Disappearing giants: a review of threats to freshwater megafauna. WIREs Water: 2017, e1208.

[5] Dudgeon D. Asian river fishes in the Anthropocene: threats and conservation challenges in an era of rapid environmental change. J. Fish Bio. 2011; 79: 1487–1524. 22136237 10.1111/j.1095-8649.2011.03086.x

[6] Strayer DL, Dudgeon D. Freshwater biodiversity conservation: recent progress and future challenges. J. N. Amer. Bent. Soc. 2010; 29: 344–358.

[7] Bellemain E, Harmony P, Gray TNE, Guegan F, Valentini A, Miaud C. et al Trails of river monsters: Detecting critically endangered Mekong giant catfish *Pangasianodon gigas* using environmental DNA. G. Ecol Cons. 2016; 7:148–156.

[8] Hogan ZS, Moyle PB, May B, Zanden MJV, Baird IG. The imperiled giants of the Mekong. Amer. Sci. 2004; 92:228–237

[9] Zarfl C, Lumsdon AE, Berlekamp J, Tydecks L, Tockner K. A global boom in hydropower dam construction. Aqu. Sci. 2015; 77:161–170.

[10] Dudgeon D. Large-Scale Hydrological Changes in Tropical Asia: Prospects for Riverine Biodiversity. BioScience 2000; 50: 793–806

[11] Ferguson JW, Healey M, Dugan P, Barlow C. Potential effects of dams on migratory fish in the Mekong River: Lessons from salmon in the Fraser and Columbia Rivers. Env. Manag. 2011; 47:141–159. 10.1007/s00267-010-9563-6 20924582

[12] Ziegler AD, Petney TN, Grundy-Warr C, Andrews RH, Baird IG, Wasson RJ. Dams and Disease Triggers on the Lower Mekong River. PLoS Negl Trop Dis 2013; 7: e2166 doi: 10.1371/journal.pntd.0002166 23853695 10.1371/journal.pntd.0002166 PMC3682813

[13] Ziv G, Baran E, Nam S, Rodríguez-Iturbe I, Levin SA. Trading-off fish biodiversity, food security, and hydropower in the Mekong River Basin. PNAS 2012; 109: 5609–5614. doi: 10.1073/pnas.1201423109 22393001 10.1073/pnas.1201423109 PMC3326487

[14] Xu J, Grumbine RE, Shrestha A, Eriksson M, Yang X, Wang YUN, Wilkes A. The melting Himalayas: cascading effects of climate change on water, biodiversity, and livelihoods. Cons. Bio 2009: 23:520–530. 10.1111/j.1523-1739.2009.01237.x 22748090

[15] Lutz AF, Immerzeel WW, Shrestha AB, Bierkens MFP. Consistent increase in High Asia's runoff due to increasing glacier melt and precipitation. Nature Climate Change 2014: 4: 587–592.

[16] Minh NH, Minh TB, Kajiwara N, Kunisue T, Iwata H, Viet PH, Tu NPC, Tuyen BC, Tanabe S. Pollution sources and occurrences of selected persistent organic pollutants (POPs) in sediments of the Mekong River delta, South Vietnam. Chemosphere 2007 67:1794–1801. doi: 10.1016/j.chemosphere.2006.05.144 17223174 10.1016/j.chemosphere.2006.05.144

[17] Neef A, Touch S, Chiengthong J. The politics and ethics of land concessions in rural Cambodia. J. Agri. Env. Ethics 2013: 26:1085–1103

[18] Yuan PA, Wei G, Cunningham AA, Li S, Chen S, Milner-Gulland EJ et al Using local ecological knowledge to assess the status of the Critically Endangered Chinese giant salamander *Andrias davidianus* in Guizhou Province, China. Oryx 2015; 50:257–264.

[19] Turvey ST., Risley CL, Moore JE, Barrett LA, Yujiang H, Xiujiang Z et al, Can local ecological knowledge be used to assess status and extinction drivers in a threatened freshwater cetacean? Biol. Cons. 2013; 157: 352–360.

[20] Anadón JD, Giménez A, Ballestar R, Pérez I. Evaluation of local ecological knowledge as a method for collecting extensive data on animal abundance. Cons. Biol 2009; 23: 617–625 10.1111/j.1523-1739.2008.01145.x 19183211

[21] Parry L, Peres CA. Evaluating the use of local ecological knowledge to monitor hunted tropical-forest wildlife over large spatial scales. Ecol. Soc. 2015; 20:3

[22] Turvey ST, Trung CT, Quyet VD, Nhu HV, Thoai DV, Tuan VCA, Hoa DT et al Interview‐based sighting histories can inform regional conservation prioritization for highly threatened cryptic species. J. App. Eco. 2015; 52: 422–433. 10.1111/1365-2664.12382 PMC4407913 25926709

[23] Drew JA. Use of traditional ecological knowledge in marine conservation. Con. Bio. 2005; 19: 1286–1293.

[24] Zukowski S, Curtis A, Watts RJ. Using fisher local ecological knowledge to improve management: the Murray crayfish in Australia. Fis. Res. 2011; 110: 120–127

[25] Baird IG. The Don Sahong Dam: Potential Impacts on regional fish migrations, livelihoods, and human health. Cr. As. Stu. 2011; 43: 211–235.

[26] Roberts T R, Baird IG. Traditional fisheries and fish ecology on the Mekong River at Khone Waterfalls in southern Laos. N. His. B. Siam. Soc. 1995; 43:219–262.

[27] Baird IG. Strength in diversity: fish sanctuaries and deep‐water pools in Lao PDR. Fish. Mang. Ecol. 2006; 13: 1–8.

[28] Baird IG. *Probarbus jullieni* and *Probarbus labeamajor*: the management and conservation of two of the largest fish species in the Mekong River in southern Laos. Aq. cons. mar. fresh. eco. 2006; 16: 517–532.

[29] Navarro D *lsr: Companion to "Learning Statistics with R* https://rdrr.io/cran/lsr/man/lsr-package.html Accessed 6/21/2017

[30] R Core Team. R: A language and environment for statistical computing R Foundation for Statistical Computing, Vienna, Austria ISBN 3-900051-07-0. 2011. URL http://www.R-project.org/ Accessed 1/5/2015

[31] Poulsen AF, Valbo-Jørgensen J. Fish migrations and spawning habits in the Mekong mainstream–a survey using local knowledge (basin-wide) *Assessment of Mekong fisheries*: *fish migrations and spawning and the impact of water management component*. 2000 Vientiane: Mekong River Commission.

[32] Turvey ST, Barrett LA, Yujiang HAO, Lei Z, Xinqiao Z, Xianyan W et al Rapidly shifting baselines in Yangtze fishing communities and local memory of extinct species. Cons. Bio. 2010: 24; 778–787. 10.1111/j.1523-1739.2009.01395.x 20067488

[33] Huntington H.P. Using traditional ecological knowledge in science: methods and applications. Ecol. Appl. 2000:10:1270–1274

[34] Agrawal A. Indigenous and scientific knowledge: some critical comments. Antropologi Indonesia. 2014 7 16.

[35] Baird IG. Conducting rapid biology-based assessments using local ecological knowledge. Natural History Bulletin of the Siam Society. 2006;54:167–75.

[36] Wilkie P, Saridan A. The limitations of vernacular names in an inventory study, Central Kalimantan, Indonesia. Biodiversity and Conservation. 1999: 8:1457–67.

[37] Mohd-Azlan J, Belant JL, Meijaard E. Can secondary information inform about population trends of carnivores in Borneo?. Raffles Bulletin of Zoology. 2013(Suppl. 28):1–8.

[38] Zhao M, Brofeldt S, Li Q, Xu J, Danielsen F, Læssøe SB, Poulsen MK, Gottlieb A, Maxwell JF, Theilade I. Can Community Members Identify Tropical Tree Species for REDD+ Carbon and Biodiversity Measurements?. PloS one. 2016 11(11):e0152061 doi: 10.1371/journal.pone.0152061 27814370 10.1371/journal.pone.0152061 PMC5096847

[39] Olden JD, Hogan ZS, Zanden M. Small fish, big fish, red fish, blue fish: size‐biased extinction risk of the world's freshwater and marine fishes. Glob. Eco. Biog. 2007; 16: 694–701.

