# Supplementary material for: Using local ecological knowledge to monitor threatened Mekong megafauna in Lao PDR
Source: PLoS One. 2017 Aug 18;12(8):e0183247. doi: 10.1371/journal.pone.0183247 (PMC5562319; doi:10.1371/journal.pone.0183247)
Supplement: S1 Table — (DOC) [file pone.0183247.s001.doc]

**S1 Table**

| **Survey Village** | **Interviewee Age** | **Years spent fishing** | **Hours spent fishing / week** |
| --- | --- | --- | --- |
| Donphapeng | 43 ± 12 (22-65) | 29 ± 11 (12-50) | 28 ± 9 (10-49) |
| Hangsadam | 41 ± 8 (28-53) | 26 ± 11 (4-47) | 39 ± 13 (16-70) |
| Lopadikhonnoi | 43 ± 9 (25-57) | 29 ± 11 (10-46) | 24 ± 10 (14-42) |
| Nadi | 39 ± 10 (23-60) | 22 ± 12 (9-50 | 18 ± 6 (11-28) |
| Veunkhaen | 50 ± 11 (25-73) | 26 ± 14 (5-58) | 14 ± 9 (4-42) |
| Veun | 50 ± 9(35-62) | 28 ± 12 (4-40) | 14 ± 10 (4-49) |
| All interviewees | 44 ± 11 (22-73) | 27 ± 12 (4-58) | 23 ± 13 (4-70) |

S1 Table. Mean ± SD and range (in parenthesis) age, years fishing and weekly fishing hours from interviewed fishermen in six survey villages in Siphandone, Lao PDR
