# Supplementary material for: Using local ecological knowledge to monitor threatened Mekong megafauna in Lao PDR
Source: PLoS One. 2017 Aug 18;12(8):e0183247. doi: 10.1371/journal.pone.0183247 (PMC5562319; doi:10.1371/journal.pone.0183247)
Supplement: S2 Table — Number of interviewees who provided last catch dates used for analysis indicated including number, in parenthesis, providing a month and year of last capture. (DOC) [file pone.0183247.s002.doc]

**S2 Table**

| **Species** | **Knowledge by interviewees (%)** | **Capture by interviewees (%)** | **Last capture dates (including month) provided** |
| --- | --- | --- | --- |
| *Pangasianodon gigas* | 41 (34) | 24 (20) | 19 (13) |
| *Pangasius sanitwongsei* | 56 (47) | 43 (36) | 34 (20) |
| *Himantura polylepis* | 108 (90) | 104 (87) | 93 (69) |
| *Dasyatis laosensis* | 101 (84) | 99 (83) | 85 (68) |
| *Probarbus spp.* | 115 (96) | 114 (95) | 106 (101) |
| *Bangana behri* | 113 (94) | 110 (92) | 102 (94) |
| *Hemibagrus spilopterus* | 119 (99) | 118 (98) | 115 (109) |
| *Barbonymus gonionotus* | 118 (98) | 117 (98) | 112 (105) |

S2 Table. Knowledge (number of interviewees with % of interviewees in parenthesis) and previous capture experience (number of interviewees with % of interviewees in parenthesis) of focal species of Mekong freshwater fish from 120 interviewed fishermen in Siphandone, Lao PDR. Number of interviewees who provided last catch dates used for analysis indicated including number, in parenthesis, providing a month and year of last capture.
