# Supplementary material for: Using local ecological knowledge to monitor threatened Mekong megafauna in Lao PDR
Source: PLoS One. 2017 Aug 18;12(8):e0183247. doi: 10.1371/journal.pone.0183247 (PMC5562319; doi:10.1371/journal.pone.0183247)
Supplement: S3 Table — (DOC) [file pone.0183247.s003.doc]

**S3 Table**

| **Survey Village** | *Pangasianodon gigas* | | *Pangasius sanitwongsei* | |
| --- | --- | --- | --- | --- |
|  | **Knowledge by interviewees (%)** | **Capture by interviewees (%)** | **Knowledge by interviewees (%)** | **Capture by interviewees (%)** |
| Donphapeng | 4 (20) | 4 (20) | 7 (35) | 6 (30) |
| Hangsadam | 13 (65) | 13 (65) | 14 (70) | 13 (65) |
| Lopadikhonnoi | 2 (10) | 1 (5) | 3 (15) | 3 (15) |
| Nadi | 11 (55) | 3 (15) | 16 (80) | 11 (55) |
| Veunkhaen | 6 (30) | 2 (10) | 10 (50) | 7 (35) |
| Veun | 5 (25) | 1 (5) | 6 (30) | 3 (15) |

S3 Table. Knowledge (number of interviewees with % of interviewees in parenthesis) and previous capture experience (number of interviewees with % of interviewees in parenthesis) of *Pangasianodon gigas* and *Pangasius sanitwongsei* by interviewed fishermen in six survey villages (n=20 in each village) in Siphandone, Lao PD
