# Supplementary material for: Using local ecological knowledge to monitor threatened Mekong megafauna in Lao PDR
Source: PLoS One. 2017 Aug 18;12(8):e0183247. doi: 10.1371/journal.pone.0183247 (PMC5562319; doi:10.1371/journal.pone.0183247)
Supplement: S4 Table — (DOC) [file pone.0183247.s004.doc]

**S4 Table**

**Translated interview form used for the study**

Survey Date:…………............., Interviewer:……………………………., Age:…..….., Position: …………………...., Ethnic:……………, Occupation: ………………………………., Village:………………………………, District:………………........., Province:………....…….

1. **Fisheries General Information**
2. How many years have you been fishing?

…………………………………………………………………………………

1. What kind of fishing gears do you use? (List all if more one type) (Mark ✔ In the bank)

Gill net,…………Gas net……….Hook Line………..Horizontal Cylinder trap………. Horizontal Cylinder trap …………Triangular Scoop Net………, Drop Door Traps……….. Filtering Traps………., Filtering Trap……….Box Funnel Net…........Door Trap………Upright Basket Trap ………, Upright Filtering Trap ………., and Other:…. ……….…………………………………………………………………………...……… … …………………………………………………...

1. Where do you like to fish ? Why?

…………………………………………………………………………………...…………………………………………………………………………...……………………………………………………………………………………...……

1. Size dimension of fishing gear as gill net, net and other? Net length? Net width?

…………………………………………………………………………………...…………………………………………………………………………...……………………………………………………………………………………...……

1. What is the type of commonly fishing gear used? How long you use for each. If you change your fishing gear, please tell us the reason why?

………………………………………………………………...………………………………………………………………………………………………………………...…………………………………………………………………...……

1. What is the commonest type of fishing gear used in your village?

…………………………………………………………………………………...…………………………………………………………………………...……………………………………………………………………………………...……

1. How many day a week do you like to fish. And how many hour a day do you fish?

…………………………………………………………………………………...…………

1. What time do you like to go for fishing ( during the day or night time )?

…………………………………………………………………………………...…………………………………………………………………………...……………………………………………………………………………………...……

1. What months/season do you get the most number of caught-fish? Do you know why?…………………………………………………………………………………...…………………………………………………………………………...……………………………………………………………………………………...……
2. What months/season do you get the smallest number of caught-fish? Do you know why?

…………………………………………………………………………………...…………………………………………………………………………...……………………………………………………………………………………...……

1. What job do you do during the fishing ban season?

…………………………………………………………………………………...…………………………………………………………………………...……………………………………………………………………………………...……

1. Most of your caught-fish are for sell or consume? If for sell- where do you sell it to (Do you sell it in the village, to the district, or to the province ) How much it is? …………………………………………………………………………………...…………………………………………………………………………...……………………………

………………………………………………………...……

1. Did you ever get caught these fishes?
2. Pa Beuk, *Pangasianodon gigas*
3. PaFa Lay, *Himantura polylepis*
4. Pa leum, *Pangasius sanitwongsei*
5. Pa Eun Ta Deang, *Probarbus jullieni*
6. Pa Wa Houa Nano, *Banga behri*
7. Pa Kot *Hemibagrus nemurus*
8. Pa Pak, *Barbonymus gonionotus*
9. Do you want your children or grandchildren to be fisherman? Generally, do you think it is a good job? If yes, Why?

………………………………………………………………...……………………………………………………………………………………………...……………………………………………………………………………………...…

1. **Species Information**

List of species:……………………………………. (Mark 1 in 7 fish species of interviewing)?

1. Pa Beuk, *Pangasianodon gigas*
2. PaFa Lay, *Himantura polylepis*
3. Pa leum, *Pangasius sanitwongsei*
4. Pa Eun Ta Deang, *Probarbus jullieni*
5. Pa Wa Houa Nano, *Banga behri*
6. Pa Kot *Hemibagrus nemurus*
7. Pa Pak, *Barbonymus gonionotus*
8. Do you know this species?

……………………………..………………………………………………………………

1. Is this species found recently in this region? What is evidences (Photos and other….)?

……………………………..………………………………………………………………………………......……………………………………………………………………………………….………………………………………………

1. Have you seen this species in general? How many time a year, How many of them? Where?

……………………………..………………………………………………………………………………......…………………………………………………………………………

1. How many times have you ever seen this species in your life? When is the last time you saw this species? Where? What month/year last seen? Where? How much? How was it caught? …………………………………………………………......…………………………………………………………………………………………......……………………………………………………………………………………….……………………………………………
2. Where do you usually find this species? Do you know how it is appear (in a group, individual, in a deep pool, Rapid river flow? and other……………?

………………………………………………………………………………………………………………………………………………………………………………………………………………………………………………………

1. Do you know what kind of food do the species like to eat most and in general? ………………………………………………………………………………………………………………………………………………………………………………………………………………………………………………………
2. In what time/season can you see this species? ………………………………………………………………………………………………………………………………………………………………………………………………………………………………………………………
3. In general. What months/season do you mostly get caught? ………………………………………………………………………………………………………………………………………………………………………………………………………………………………………………………
4. Fish breeding season?……………………………………Immigration season ? ………..……………………………………………………… ………………….... … ……………………………………………………………
5. Do you know the trend seeing of this fish population?

| Year | Recent | 2 yaer ago | 5 year ago | 10 year ago | 15 year ago | 20 year ago | Other comments |
| --- | --- | --- | --- | --- | --- | --- | --- |
| ……….. |  |  |  |  |  |  |  |

1 – Most, 2 - Mid, 3 - Least, 4 – Disappeared

1. It is a dangerous species?

……………………………………………………………………………………………

1. Have you ever seen or heard that people to get caught this specie? If yes, how it was caught?…………………………………………………………………………………………………………………………………………………………………………………………………………………………………………………………………
2. How long do you think this species disappeared? …………………………………………………………………………………………………………………………………………………………………………………………………………………………………………………………………
3. Why did you think this species disappeared?

…………………………………………………………………………………………………………………………………………………………………………………………………………………………………………………………………

1. What is your suggestion on fish species conservation in order to prevent ending its life …………………………………………………………………………………………………………………………………………………………………………………………………………………………………………………………………
